# The Historical Evolution of the Role of Vegetation in the Enhancement and Conservation of Archaeological Sites: A Landscape Architecture Perspective Focused Mainly on Cases from Italy and Greece

**DOI:** 10.3390/plants14152302

**Published:** 2025-07-25

**Authors:** Electra Kanellou, Maria Papafotiou

**Affiliations:** Laboratory of Floriculture and Landscape Architecture, Department of Crop Science, Agricultural University of Athens, Iera Odos 75, 11855 Athens, Greece; ilekanellou@aua.gr

**Keywords:** vegetation management, archaeological conservation, historical landscape, cultural heritage, ancient monument restoration, landscape design, vegetation design, ornamental vegetation

## Abstract

Vegetation plays a multifaceted role in the enhancement and conservation of archaeological sites, functioning not only as an aesthetic element but also as a core component of landscape architecture practice. This review traces the historical evolution of vegetation management, though the lens of landscape architecture, highlighting its potential as a design and planning tool for historical interpretation and sustainable integration of heritage sites into broader contexts. From Romantic landscaping ideals to modern interdisciplinary conservation frameworks, the review draws on key milestones such as the Athens and Venice Charters, and examines case studies like Rome’s Passeggiata Archeologica, the Acropolis slopes, Ruffenhofen Park, and Campo Lameiro. These examples illustrate how landscape architectural approaches can use vegetation to reconstruct lost architectural forms, enhance visitor engagement, and provide ecosystem functions. The article also addresses challenges related to historical authenticity, species selection, and ecological performance, arguing for future strategies that integrate archaeological sites into dynamic, living heritage systems, through collaborative, ecologically informed design.

## 1. Introduction

The restoration and conservation of ancient monuments is globally recognised as a fundamental societal value, safeguarded by national and international laws and institutions such as ICOMOS and UNESCO, primarily with the aim of preserving memory and knowledge for future generations [1]. The principles that guide monument restoration are not fixed or self-evident truths, but the result of complex ethical, political, and emotional processes that have evolved over more than two centuries and continue to evolve today [2].

Understanding this historical evolution not only of restoration and conservation practices but also of the very concepts and objectives of protection and preservation is essential. This perspective offers critical insight into why ancient monuments have acquired significance and why they are managed within a complex and multifaceted framework today [3].

Currently, integrated conservation positions monuments as active elements in spatial, cultural, educational, and environmental planning, advocating for an interdisciplinary approach that encompasses archaeology, architecture, urban planning, ecology, and education. Particular emphasis is placed on education and interpretation, which are key processes that enhance the accessibility and relevance of heritage sites to a broader audience [3].

Crucially, archaeological sites are inextricably linked to their surrounding landscape, and landscape architecture contributes decisively to shaping this landscape. For the purposes of this review, an archaeological site refers to any location that contains material evidence of past human activity that has been investigated using the discipline of archaeology [4]. An archaeological landscape refers not only to the large-scale spatial organisation of heritage elements, but also to how visitors perceive, experience, and interpret their surroundings, an understanding that calls for an interdisciplinary lens [5].

Landscape architecture, as defined by the International Federation of Landscape Architects (IFLA), is concerned with the creation, conservation, and management of landscapes from wild nature to urban, through both design and planning interventions. It addresses the relationship between people, place, and nature, integrating ecological, cultural, and social dimensions across spatial and temporal scales [6]. A basic component of landscape architecture is vegetation, which plays a vital role both in shaping the identity of the archaeological landscape and in supporting conservation and interpretation strategies.

Within the framework of heritage conservation, vegetation as a component of landscape architecture plays an important and multifaceted role, not only in conservation, education, aesthetic enhancement, and improving site legibility and visitor experience [7], but also in contributing to the socio-economic development of the area through tourism, job creation, investment opportunities, and the strengthening of local social ties [8].

Moreover, amid growing interest in sustainability and climate resilience, vegetation of archaeological sites plays a vital role in preserving biodiversity [9,10] and supporting ecologically informed conservation approaches [11,12,13], delivering essential ecosystem services and enhancing the resilience of archaeological sites [14,15]. The increasing attention to intangible heritage and the “spirit of place” in conservation theory further highlights the cultural significance of landscape elements such as vegetation.

From the very beginning, vegetation has been an integral part of the evolving framework of ancient monument conservation, with its role and value also undergoing significant transformation in response to shifting values, aesthetic ideals, and environmental priorities [16].

The aim of this paper is to examine the evolving role of vegetation as a key element in the conservation and enhancement of ancient monuments, exploring historical developments and contemporary approaches, through the lens of landscape architecture. The paper examines how vegetation, beyond its aesthetic contribution, can play an active role in the conservation and presentation of archaeological sites, aligning with international heritage principles. This is especially relevant to Greece which has 3100 archaeological sites [17], which necessitate sustainable approaches that respond to both the conservation needs of monuments and their environmental surroundings.

This paper contributes to bridging the gap between aesthetic, ecological, and cultural heritage perspectives and critical examination of charters and international conservation principles, showing how they have created space for vegetation’s active role and encouraging interdisciplinary approaches that include landscape architecture and ecology.

The paper reviews the theoretical framework of monument restoration, which began to take shape in Europe during the Enlightenment and evolved through landmark moments such as the Athens and Venice Charters. Through case studies from Greece, Italy, and other regions, it illustrates how this framework has been creatively applied in the selection and use of vegetation to enhance, protect, and interpret ancient monuments.

## 2. Methodology

This review adopts a landscape architecture perspective to examine the evolving role of vegetation in the conservation and enhancement of archaeological sites, with a regional focus mainly on Italy and Greece. The research approach emphasises a cross-disciplinary integration of design theory, heritage conservation principles, and ecological planning, aiming to critically evaluate how vegetation has been historically and contemporarily deployed as a spatial, ecological, and interpretive element in archaeological landscapes.

The central research question explores how vegetation has been conceptualised, managed, and integrated within archaeological sites over time, particularly through practices aligned with landscape architecture, including site design, visual composition, spatial narration, ecological restoration, and visitor experience enhancement.

The primary sources include international charters and declarations—such as the Athens Charter (1931) [18], Venice Charter (1964) [19], the Granada Convention (1985) [20], the Charter for the Interpretation and Presentation of Cultural Heritage Sites (2008) [21], and the Québec Declaration (2008) [22]—which collectively define the ethical, technical, and philosophical framework of historical and contemporary conservation. These documents were analysed through a landscape architecture lens to extract principles relevant to the treatment of vegetation in heritage contexts. Particular attention was paid to concepts such as authenticity, reversibility, minimal intervention, landscape integration, and didactic function, assessing how these intersect with landscape design and planning methodologies.

In addition to international policy texts, the study draws on scholarly publications that engage with vegetation through ecological, design-based, or planning frameworks within cultural heritage contexts. A comprehensive literature search was conducted using academic databases including Web of Science, Scopus, JSTOR, ScienceDirect, and Google Scholar. The search strategy employed keywords such as vegetation in archaeological conservation and heritage sites, archaeological parks, heritage vegetation management, archaeological site landscape design and cultural landscape and ecological planning.

Case studies were selected based on three criteria: (a) documented integration of vegetation in site interpretation or restoration; (b) recognition within academic or official heritage literature; and (c) alignment with internationally accepted conservation frameworks. While the primary focus is on Italy and Greece, comparative examples from other regions are included to illustrate broader design strategies and management models.

The findings were synthesised through a multidisciplinary interpretive framework that centres landscape architecture, while incorporating insights from heritage studies, ecological restoration, and conservation theory. This approach allows for a nuanced understanding of vegetation not merely as a background or technical element, but as an active agent in the spatial, ecological, and cultural design of archaeological landscapes. The review concludes by outlining future directions for sustainable, community-engaged, and ecologically responsive vegetation planning within the field of archaeological site conservation.

## 3. Historical Evolution

### 3.1. Early Developments (18th to 19th Century)

The concept of monument preservation, as we understand it today, began to take shape in Europe toward the end of the 18th century, rooted in the intellectual movements of the Enlightenment. These currents laid the groundwork for early restoration practices by promoting the idea of historical artefacts as valuable and meaningful. At the same time, the development of historical science enabled monuments to be studied more analytically, as sources of evidence rather than merely aesthetic objects [3] (pp. 75–77). In the 19th century, the formation of nation-states and the rise of nationalism reinforced the importance of preserving monuments, not only as relics of the past but as symbols of national identity and vehicles for shaping collective memory [23]. Influential figures such as John Ruskin and Viollet-le-Duc embodied two contrasting approaches: the former emphasising the preservation of material authenticity, the latter favouring stylistic restoration [2] (pp. 137–212). These tensions, alongside growing institutional support across Europe, helped define the monument as a heritage object meant to serve both present and future generations [3] (pp. 75–77).

The case of Rome occupies a particularly prominent place in the study of archaeological landscapes and conservation practices. Rome serves as a universal reference point for the global imagination of ancient urbanism, cultural memory, and the meaning of heritage itself [24]. From the Grand Tour era onward, its ruins have defined key principles of monument preservation, archaeological excavation, and landscape integration. Consequently, examples from Rome are especially valuable for exploring how vegetation has historically interacted with archaeological sites, and how these interactions continue to inform contemporary conservation practices.

One of the earliest systematic monument restoration efforts took place in Rome during the early 19th century (1800–1830), particularly during the Napoleonic occupation (1809–1814). A bold initiative aimed to consolidate the city’s central ruins into a unified archaeological park—Il Jardin du Capitol—with vegetation playing a central role in enhancing both the visibility and symbolic resonance of the monuments [16,24,25]. In 1811, papal architects Giuseppe Camporesi and Giuseppe Valadier were tasked with designing the park, envisioning a romantic landscape in the English picturesque style. Their plan, however, failed to satisfy the French authorities [2,26]. Romanticism developed in contrast to the ideological foundations of the Enlightenment, placing nature and the natural landscape at the centre, attributing intrinsic value to it [27]. Two years later, Louis Berthault proposed a more eclectic design that blended formal French gardens with picturesque elements, treating the monuments as theatrical scenery. Political instability prevented either plan from being implemented. Still, the core idea of a unified archaeological park shaped by vegetation endured and would later influence the creation of the Passeggiata Archeologica (Archaeological Walk) almost a century later [24,25].

This episode illustrates a key philosophical tension that continues to shape conservation discourse. Camporesi and Valadier emphasised archaeological integrity, insisting on thorough excavation before any landscape intervention. Berthault, by contrast, prioritised aesthetic effect, treating monuments as picturesque elements in a curated setting, even at the expense of historical accuracy. The contrast between philological rigour and evocative scenography remains a central issue in how archaeological landscapes are designed and understood today [25]. These early developments laid the groundwork for later conservation ideologies, introducing both the potential and the challenges of integrating vegetation into the presentation of archaeological sites.

These debates would later contrast sharply with the late 19th-century trend toward purifying archaeological sites by removing vegetation altogether, as seen in Pietro Rosa’s clearing campaigns at the Colosseum, which prioritised exposing the monument’s structure over maintaining its romantic character [28].

### 3.2. Landmark Moments: Early 20th Century and the Athens Charter

Following Rome’s designation as the capital of unified Italy in 1870, the archaeological landscape became a battleground for constructing national identity, with planners emphasising the sanctity of ancient ruins as symbols of the newly formed state [28]. In the early 20th century, Rome’s Passeggiata Archeologica marked a turning point in archaeological site presentation.

This large-scale project brought together major landmarks—including the Roman Forum, Palatine Hill, Colosseum, Forum Boarium, and Baths of Caracalla—through a continuous landscape intervention. It involved significant expropriations and demolitions, along with the deliberate planting of vegetation based on local plants [16]. This project represented one of the earliest examples of a deliberately planned archaeological park, integrating monuments and landscape into a unified vision of national heritage. The establishment of the Passeggiata Archeologica was not merely an aesthetic enterprise, but also a political and ideological gesture that affirmed Italy’s historical legitimacy through its ancient past [28].

Between 1899 and 1922, architect Giacomo Boni led a systematic effort to create harmony between ancient structures and natural elements. He selected plant species that suited not only the historical character of the ruins but also the environmental and ecological context [25].

Boni, as Ruskin’s follower, supported the Anti-Restoration Movement and rejected intrusive interventions that radically alter the form and state of preservation of monuments, as they removed material authenticity and surface ageing. Instead, he advocated for anastylosis, i.e., the careful reassembly of original architectural elements [16]. For Boni, nature played a vital role in enhancing the evocative power of ruins [29]. As he wrote in 1913, “Nature, reclaiming materials that man has removed, acts harmoniously, granting ruins the ability to evoke intellectual representation. This is where their charm and beauty originate” [30]. Boni’s pioneering approach did not stop at aesthetic enhancement; he introduced stratigraphic excavation methods and emphasised the scientific importance of contextual archaeological recording. His work bridged Romantic ideals with a meticulous scientific methodology, making him a precursor to modern archaeological conservation [28].

Boni offered a visionary and practical perspective on the role of vegetation in archaeological landscapes [30]. He saw flora not only as a protective element but as a means of education and aesthetic enrichment, provided it was carefully managed. He wrote, “I would like to enrich the flora of the Palatine. I wish to instil the educational influence derived from the love and respect for plants, which many visitors seem to greatly need” [30].

He also noted the protective value of grasses in forming a thin organic layer over ruins, shielding them from heat and frost through root networks. To safeguard vulnerable masonry, he proposed spreading cocciopesto mortar topped with soil and drought-tolerant species like lippia (*Phylla nodiflora*). Boni also warned against invasive trees like black locust (*Robinia pseudoacacia*) and tree of heaven (*Ailanthus altissima*), urging that each country preserve its native monumental flora [30].

Boni’s approach extended beyond theory into detailed practice. Collaborating with geologists and naturalists, he documented the native flora and selected plantings for their visual, ecological, and historical relevance. In the Roman Forum, he used boxwood (*Buxus sempervirens*), juniper (*Juniperus* sp.), sedum (*Sedum* sp.), ice plants (*Mesembrianthemum* sp.), roses (*Rosa* spp.), and ivy (*Hedera helix*) to delineate zones, conceal intrusive elements, stabilise soils, and shield ancient surfaces. To evoke the site’s classical past, he also planted species known from ancient sources, such as cypress (*Cupressus* spp.), laurel (*Laurus nobilis*), myrtle (*Myrtus communis*), pine (*Pinus* spp.), and grapevines (*Vitis* spp.), thereby constructing a romanticised yet respectful landscape [16].

Boni’s work laid the foundation for future theoretical models of archaeological landscaping. Grounded in careful field observation and ecological awareness, his designs demonstrated how vegetation could serve both protective and interpretive roles. Many of his interventions remain visible in Rome’s historic centre today [25].

The planning of the Passeggiata Archeologica was also strongly influenced by broader cultural movements and individuals beyond Boni. Maria Ponti Pasolini, an early advocate for the integration of vegetation into urban and archaeological landscapes, played a pivotal role in shaping public discourse around the “spirit of place”. Together with figures like Gustavo Giovannoni, Pasolini promoted the view—rooted in Anglo-Saxon landscape ideals—that ruins and vegetation form an inseparable aesthetic and cultural unity. The AACAR (Artistic Association of Architectural Connoisseurs) became a central platform for advancing these ideas, emphasising that the conservation of monuments should preserve both material remains and their vegetated, historical environments [29].

During the same period (1898–1905), Marquis Curzon of Kedleston, serving as Viceroy of India, initiated significant restoration efforts, most notably at the Taj Mahal. His work included structural reinforcements and the replanting of flowers and trees in the site’s excavated gardens, reflecting a growing recognition of vegetation’s role in the presentation of monuments [2] (p. 275).

A century after the first large-scale conservation and restoration initiative, the Athens Charter (1931) marked a major milestone as the first international framework for monument restoration. Among its key contributions was an emphasis on the surrounding environment of monuments. At the Athens Conference, experts agreed that vegetation could meaningfully enhance archaeological sites. Horta, representing the Royal Academy of Belgium, suggested that carefully positioned plantings could symbolically evoke lost architectural forms. Lensi of Florence’s Fine Arts Office supported the use of scale-appropriate plantings as aesthetic complements to monuments. Greek archaeologist Oikonomos proposed isolating sites from urban encroachment with a 500 m buffer zone, where vegetation would act as both transition and restoration, reconnecting the site to its historical landscape [16].

Article III of the Athens Charter formalised these discussions, emphasising that the surroundings of monuments require special attention [18]. It called for landscape design plans that include ornamental vegetation chosen to preserve and enhance the character of each site. The Athens Charter was among the first to explicitly position vegetation as both a design and conservation strategy, linking environmental enhancement to the broader goal of cultural legibility [16].

The interwar period saw the rise of mass tourism to archaeological sites across Europe, prompting new approaches to monument restoration. This shift reflected growing pressure to adapt conservation to the demands of a mass public, a transition from scientific excavation to public-oriented scenography [31]. Archaeologists such as Guido Calza and Amedeo Maiuri, working in Ostia and Pompeii, respectively, sought to balance preservation with public interpretation. Their goal was to protect the sites while effectively communicating their historical significance to a growing audience [3,31] (pp. 194–203). As part of this strategy, restorations focused on key features of Roman houses—roofs, atriums, and peristyles—while decorative elements and furnishings were arranged in situ to recreate scenes of ancient domestic life. Maiuri viewed Pompeii’s primary value as its potential to reveal everyday Roman experiences [16,32]. Interior gardens were also restored, using species and designs based on ancient descriptions of Roman horticulture [16]. These reconstructions deepened visitor immersion, offering a more tangible and educational experience of the ancient urban landscape.

In contrast to the more careful restorations in places like Pompeii, Rome experienced aggressive interventions during the 1930s, driven by a desire to glorify its imperial past. Many restorations went too far—removing later historical layers, reconstructing features arbitrarily, and conducting undocumented excavations—often at the expense of authenticity and scholarly integrity [3] (pp. 194–203). Amid this context, Antonio Muñoz, head of the Directorate of Antiquities and Fine Arts, stood out for his more nuanced approach. He supported integrating monuments into the modern city through transitional green zones and used vegetation symbolically to represent missing architectural elements. This philosophy was demonstrated in the 1934–1935 restoration of the Temple of Venus and Rome. Muñoz used *L. nobilis* to indicate the lost northern wall, recreated podium steps with *B. sempervirens* and represented missing columns with cornel trees (*Cornus mas*). He emphasised that no artificial or conjectural materials were added—only plants were used, a form of restoration that avoids physical conjecture by replacing missing architecture with symbolic plantings [16].

Muñoz also led the 1938 restoration of the Mausoleum of Augustus, originally constructed in 28 BCE and historically described as encircled by a grove of evergreen trees. Over centuries, the structure had been altered for various uses: a Renaissance garden, an 18th-century bullfighting ring, and later a concert venue. Seeking to recover its original spirit, Muñoz removed later additions, including a modern roof, and planted cypress trees (*Cupressus sempervirens*) atop the restored structure to recreate the ancient grove [16]. The result remains visible today as a rare example of symbolic restoration through vegetation.

The early 20th century saw vegetation emerge not only as an aesthetic or protective element but also as a symbolic and interpretive tool in archaeological restoration. Projects like those led by Boni and Muñoz exemplify how plants could express historical meaning and enhance the cultural presence of ancient sites.

### 3.3. Post-War Developments and the Venice Charter

In the aftermath of World War II, the rebuilding of European cultural heritage sparked major debates around monument restoration. In Italy, the limitations of existing frameworks led to the rise of the critical restoration school and the concept of creative restoration. This approach prioritised the artistic value of monuments and allowed for carefully considered reconstructions and additions, only when the existing structure remained sufficiently intact. If not, no intervention was made, respecting the irreplaceability of the original artistic act. The goal was to make monuments legible and functional while maintaining historical integrity. At the same time, restoration theory broadened to consider the urban scale, advocating for the integration of historic centres into modern city life. This approach sought to prevent the isolation or abandonment of heritage sites by embedding them in the social and functional fabric of the contemporary city [3] (pp. 209–228).

These evolving ideas—especially those championed by Cesare Brandi—were consolidated in the Venice Charter of 1964, which became a foundational international document for heritage conservation [19]. The Charter redefined monuments as not only individual buildings but also broader cultural sites shaped by history and society (Article 1). It also emphasised that restoration is a scientific discipline requiring collaboration across the arts and sciences (Article 2). The Charter stressed that conservation should prioritise socially beneficial uses for monuments and ensure their continued relevance. A key principle was that “Monuments cannot be separated from their environment” (Article 6). Restoration efforts were to be grounded in evidence, halting where conjecture begins. Any necessary additions should be clearly distinguishable from the original structure (Articles 9 and 12). While reconstructions were generally excluded, anastylosis—the reassembly of original fragments—was permitted to restore structural and visual continuity. Interpretation that aids public understanding was encouraged, so long as it preserved the monument’s authenticity [19].

Two landmark projects in Athens reflected these new conservation principles through landscape design: the development of the Ancient Agora by the American School of Classical Studies and the landscaping of the Acropolis slopes by architect Dimitris Pikionis. These post-war Greek projects exemplify how the conservation philosophy outlined in the Athens and Venice Charters matured into integrated design practices, where vegetation, ruins, and modern access were balanced to create legible and meaningful archaeological spaces [3] (pp. 260–263).

As early as 1832, architects Stamatios Kleanthis and Eduard Schaubert proposed transforming the spaces between Athens’ ancient monuments into a landscaped garden with trees and lawns, envisioning a vast open-air museum of antiquity [33]. This idea was part of their broader urban plan for the newly designated capital of Greece [2] (pp. 89–91). Although the concept resurfaced intermittently in the interwar years, it remained unrealised until the mid-20th century [16].

The eventual landscaping of the Ancient Agora marked a turning point in Greek archaeological practice. Landscape architect Ralph E. Griswold, who led the project, described it as unprecedented in Greece: “Among all the excavations of ancient sites in Greece, there is no precedent for the proposed systematic landscaping of the Athenian Agora’s surroundings. It is a pioneering endeavour. It is as unique for contemporary archaeological practice, as the historical significance of the Agora itself and it will bring new interest to its ancient traditions.” [34].

The primary objective of the landscaping was to highlight the historical importance of the Agora. Vegetation was planted in areas known to have been green spaces in antiquity, while additional plantings served multiple functions: offering shade, framing monuments, marking important features, and enriching the site’s visual appeal. Near the Hephaisteion, ancient planting trenches cut into the rock were replanted with trees to recreate the garden of Hephaestus [34].

Griswold placed strong emphasis on plant diversity—incorporating trees, shrubs, herbs, and wildflowers—while prioritising native Greek species, except for a few fully acclimatised to the Attic environment [34], such as *Acacia cyanophylla*, *Brachychiton populneum*, *Eucalyptus rostrata*, *Melia azedarach*, *Robinia pseudoacacia*, *Lantana camara* and *Agave americana* [35]. Sourcing suitable plants proved difficult during the early phases. To meet the planting needs, wild flora was collected from regions around Attica. Additional plants came from state forestry nurseries and private estates [35]. Ultimately, more than 4500 plants from over 250 species were installed across the site [34]. Vathis later described the planting scheme as “suited to the Greek environment, adapted to its ecological and historical context, and effectively utilising the rich native flora” [35].

Between 1954 and 1957, architect Dimitris Pikionis undertook the landscaping of the Acropolis slopes, including the access paths to the Philopappos monument on top of Muse Hill (Philopappos Hill), opposite the Acropolis and the Church of Agios Dimitrios Loumbardiaris (Saint Demetrius the Bombardier), a Byzantine-era church at the base of Philopappos Hill. Pikionis approached the project with great sensitivity and craftsmanship, supervising every stage personally. He famously stated that he wanted to “build it himself using the hands of craftsmen” [36,37].

The design of the pathways drew inspiration from their ancient precursors and served both functional and experiential purposes, offering access while encouraging leisurely movement and contemplation [3] (pp. 260–263). Resting areas and viewpoints were integrated throughout. The most iconic aspect was the paving, created with a variety of materials including concrete, natural stones, and salvaged ceramic tiles from non-significant ancient ruins. The layout and texture of each section were composed directly on-site, emphasising improvisation and intuition [38].

Vegetation played a crucial role in Pikionis’ intervention. He planted species noted in historical records as native to the area, focusing on shrubs like *Rosa* spp., *L. nobilis*, and *M. communis*, along with both wild and cultivated olive trees (*Olea europea*) [38,39]. He also removed non-native species, believing they disrupted the natural and cultural harmony of the site [39].

For Pikionis, the Acropolis landscape embodied a deep dialogue between nature and art. In a 1940 reflection, he emphasised the transformation of natural elements into human expression: “The plastic sensitivity of materials, the infinite variety in the composition of elements that reality offers us, are gifts of nature. Nature must become art”. He praised ancient Greek architecture for integrating naturally with the land, citing the Parthenon’s foundation as carved directly into the rock, an emblem of how “the transition from Nature to Art is achieved” [40].

Pikionis’ work on the Acropolis slopes is regarded as a landmark in modern landscape architecture and is integral to the identity of the site and deeply ingrained in public memory [3] (pp. 260–263). In 1996, it was officially declared a “Historic Listed Monument” by ministerial decree, recognised as “a unique work of rare architectural value and of global interest. It is a creation of post-war architecture, and in fact by one of the most important Greek architects of the 20th century, D. Pikionis” [41].

Around the same time, a systematic tree-planting initiative took place at the archaeological site of Kerameikos, led by Judith Perlzweig-Binder of the American School of Classical Studies in Athens. In the 1950s, she landscaped the ancient cemetery near the Eridanos River to evoke the atmosphere of a classical burial ground. Drawing from ancient sources, she planted over 300 native trees, many of which held funerary associations in antiquity. The vegetation also served practical purposes, such as hiding nearby modern intrusions. Today, the German Archaeological Institute maintains the plantings in accordance with Binder’s original vision, occasionally adding new species as needed [42].

Post-war restoration theory deepened the role of vegetation in archaeological landscapes, moving beyond aesthetics to include symbolism, ecological protection, and the spirit of place. Landmark projects in Athens, Greece, such as those by Pikionis and Griswold, illustrate how plants became integral to both conservation philosophy and site experience.

## 4. Contemporary Approaches

Landscape interventions at archaeological sites are governed by established restoration principles aimed at preserving both monuments and their surroundings. The Venice Charter (1964) redefined monuments to encompass not only renowned works of art and architecture but also historic sites and cultural ensembles, while establishing core preservation methods [19]. Modern conservation is guided by ethical and technical considerations, emphasising minimal intervention, use of compatible materials, and public accessibility that ensures both clarity and safety. All interventions must align with sustainable development goals, avoid visual or physical intrusion, and be based on transparent, proportionate decision-making processes. Core principles also include interdisciplinary collaboration, reversibility of interventions, and respect for authenticity, including the *genius loci* or spirit of place [3] (pp. 322–348). The Québec Declaration defines spirit of place as the sum of a site’s tangible and intangible elements that confer meaning and identity, reinforcing the view that monuments are dynamic carriers of memory, identity, and cultural continuity [22].

Subsequent to Venice, charters such as the Amsterdam Declaration (1975) and the Granada Convention (1985) advanced the idea of integrated conservation, which situates archaeological sites within broader spatial and environmental planning [3] (pp. 208–248). This holistic model emphasises sustainability, public engagement, and educational value. It promotes heritage as a living part of contemporary life, supported by interdisciplinary collaboration and interpretive strategies—including design interventions and reconstructions—that make sites more accessible and meaningful [3] (pp. 22–26).

The Charter for the Interpretation and Presentation of Cultural Heritage Sites (2008) introduced a comprehensive set of principles for managing cultural landscapes. It emphasised enhanced access, both physical and intellectual, broader public engagement, respect for authenticity, sustainable practices, and interdisciplinary collaboration involving experts, communities and stakeholders [21].

Plants selected for archaeological contexts should reflect the historical landscape character and cultural associations of the site [43]. Sources such as art, frescoes, sculptures, ceramics, and archaeobotanical evidence provide guidance for identifying appropriate species. Roman viridaria and horti, for example, show how vegetation was arranged in antiquity and can inform contemporary reconstructions [44]. Symbolic associations from mythology also offer guidance, with many native species linked to deities, like *O. europea* to Athena, pomegranate (*Punica granatum*) to Demeter, *L. nobilis* to Apollo, and fig (*Ficus carica*) and *Vitis* spp. to Dionysus [45].

Similarly, in the peristyle garden of the House of Augustus in Rome, a full-scale (1:1) garden reconstruction used species identified from Pompeian wall paintings, such as viburnum (*Viburnum* spp.), oleander (*Nerium oleander*), *Rosa* spp., *C. sempervirens*, and quinces (*Cydonia oblonga*) [46] (pp. 364–365), combining symbolic planting and archaeological authenticity.

At the Roman villa of Eleusis, a garden restoration design was inspired by the atrium and hortus types, using species known from Roman horticulture such as *M. communis*, *B. sempervirens* and *P. granatum*. The planting aimed to enhance visitor orientation and interpretation while ensuring low maintenance. This project demonstrates how historical typologies and species selection can support both legibility and sustainability [47].

Ecological compatibility with the site’s climate, soil and microhabitats is also essential [45,48,49]. Both direct, such as root-related mechanical damage and visual obstruction from plant canopies, and indirect negative impacts, such as microclimatic changes that encourage microbial growth [44], must be carefully assessed. At Eleusis, for example, olive seedlings sprout on monument surfaces, causing damage, due to seed dispersal from nearby planted trees [50].

At the archaeological site of Olympia, a UNESCO World Heritage monument in Greece, the restoration efforts following the 2007 wildfires, which severely damaged the site, illustrate how environmentally sensitive plant selection can support both conservation and landscape recovery. Plant species selection was directed by the Central Archaeological Council of the Ministry of Culture of Greece, based on three criteria: fire resistance, historical relevance, and aesthetic integration. Drawing from local flora and ancient textual references, the plantings included species such as *Quercus coccifera*, *Q*. *ilex*, *Pinus pinea*, *C. sempervirens*, *Tilia tomentosa*, *Pistacia lentiscus*, *Vitex agnus-castus*, *Arbutus unedo*, *A*. *andrachne*, *Spartium junceum*, *Rosmarinus officinalis*, *Artemisia arborescens*, and *Satureja thymbra* [51].

Vegetation interventions, like all conservation measures, must be reversible and avoid compromising the site’s integrity. Designs should allow for future removal or modification without damaging archaeological layers. At the Giardino di Artemide in Syracuse in Italy, a contemporary garden was created around the access point to an underground Ionic temple dedicated to Artemis. The design aimed to blend spontaneous vegetation with metal and gravel elements, evoking mythological associations tied to the goddess Artemis, protector of nature. The interventions were fully reversible and preserved stratigraphy from earlier excavations [52,53,54].

Non-native and invasive species are generally unsuitable for archaeological sites. Introduced plants and modern hybrids—such as genera *Rosa*, *Acacia*, *Opuntia*, *Agave*, *Yucca*, *Eucalyptus* and *Pittosporum*—should be avoided in Mediterranean landscapes [44]. Invasive species like *A. altissima* and *R. pseudoacacia* can spread rapidly, damage monuments, and disrupt ecosystems [55,56,57].

Sustainability in conservation also calls for low-maintenance planting schemes that harmonise with the archaeological context. A study evaluating two herbaceous seed mixtures from species selected from archaeological sites in Greece showed that low-growing forbs—such as *Trifolium subterraneum*, *Medicago polymorpha*, and *Calendula arvensis*—can provide aesthetically coherent and ecologically functional vegetation cover. The findings support a model for naturalistic, low-maintenance plantings under Mediterranean conditions [58].

Collectively, these criteria ensure that vegetation becomes a meaningful component of archaeological conservation, supporting historical legibility, environmental stability, and interpretive value without compromising the authenticity or integrity of the site.

Vegetation and landscape design can function as interpretive tools that enhance the legibility of archaeological remains and foster public engagement. Through spatial delineation, symbolic plantings, and reconstructions of historical forms, vegetation bridges the gap between abstract archaeological information and tangible visitor experience.

At Ancient Messene in Peloponnese, Greece, over the last two decades, excavation, conservation, stabilisation, and partial restoration have revitalised the archaeological site. Under Professor of Classical Archaeology Petros Themelis, the theatre and stadium were restored, with reassembled original elements and reconstructing missing volumes with terrain shaping and turf covering, improving spatial comprehension without compromising authenticity. The project was awarded the Europa Nostra Prize in 2011 [59] for its exemplary integration of conservation and contemporary use, with the restored structures now hosting a wide range of public events.

Similarly, at the Teatro Romano di Gubbio in Italy, missing seating was indicated through stepped, vegetated landforms [46] (pp. 363). This approach conveyed the theatre’s original form, while avoiding the excessive use of hard materials, aligning with the conservation principles.

Other projects adopt interpretive planting as a way to communicate spatial organisation. The Ruffenhofen Roman Park and Limeseum in Germany, located along the Limes Germanicus, a UNESCO World Heritage Roman frontier complex, illustrates a large-scale interpretive planting [46] (pp. 365–367) [60]. Although the Roman structures are now subsurface, their layout remains legible. Vegetation was used to visualise these remains: hornbeam trees (*Carpinus betulus*) traced the fortification lines, interrupted by dogwoods (*Cornus* spp.) at locations corresponding to towers. Within the fort, structures were outlined using herbaceous flowering plants, while surrounding areas were covered with tightly mown turf to create clear contrast. Granary locations were planted with wheat (*Triticum spelta*), while a temple was symbolically indicated by plantings of *B. sempervirens*. The project prioritised the use of native species, enhancing local biodiversity, and tree planting was carefully planned to prevent root damage to archaeological layers. The design offers educational and recreational value, while remaining fully reversible [46] (pp. 365–367).

At the Roman house complex Domus dell’Ortaglia in Brescia, Italy, vegetation was integrated into conservation and interpretation efforts. A contemporary shelter was constructed over the site, while a rooftop garden outlines the floor plans of the Roman houses at a 1:1 scale, using hard materials and turf [61]. This landscape intervention protects the ruins and helps visitors understand the spatial layout.

Vegetation can be used symbolically to reference elements enriching the visitor sensory engagement. On the Palatine Hill in Rome in 2012, interventions in the imperial and Farnese gardens were part of the exhibition Orti e Giardini: il cuore di Roma antica. Plantings evoked key features of the nymphaea, with white- and blue-flowered species symbolising marble and water [46] (pp. 364–365).

Vegetation offers significant environmental protection for archaeological monuments. Strategically planted trees and shrubs can regulate the microclimate, providing shade and reducing temperature fluctuations. Vegetation also buffers against strong winds and limits the deposition of saline aerosols on vulnerable structures in coastal areas [44]. Technological tools such as ENVI-met modelling have shown that vegetation can improve microclimate regulation at archaeological sites, reducing stress on remains and enhancing visitor experience. Informed vegetation management can balance conservation needs with microclimate preservation, as over-reduction in vegetation may increase thermal discomfort and risk site abandonment [14,15,62].

Vegetation helps mitigate air pollution, by trapping particulates and pollutants on leaves, reducing the exposure of structural materials to harmful environmental agents [43]. Ground cover also prevents soil erosion, supporting physical stability and long-term preservation of archaeological features [44]. These ecological functions, strengthen the site’s resilience and complement conventional conservation strategies.

Vegetation interventions can restore degraded landscapes, support native ecosystems, and highlight the long-term interactions between human and natural systems. This approach aligns with heritage and environmental sustainability objectives. Recent studies highlight that the landscape surrounding archaeological sites often preserves complex, long-term interactions between natural ecosystems and human activities, reinforcing the need for conservation approaches that respect both ecological and historical dimensions [63]. Insights from the Sicani Mountains in Italy demonstrate how traditional land-use practices can maintain biodiversity and ecosystem stability over centuries [64]. Selecting an appropriate historical baseline can be a central challenge in vegetation planning [65].

The Archaeological Park of Neapolis in Syracuse integrates natural and cultural heritage, through the conservation of rare native species. *Origanum onites*, likely introduced by Greek settlers, and *Elatine gussonei,* a seasonal hydrophilous plant growing in ancient quarry cavities, reflect the site’s historic and natural legacy. An educational trail guides visitors to key habitats, linking biodiversity with heritage interpretation and deepening public engagement [7].

A recent field-based study in Greece found that spontaneous vegetation across seven archaeological sites reflected unique ecological contexts, highlighting the potential of reinforcing the site’s identity [66].

Vegetation at archaeological sites can function as a floristic conservatory, developing naturally or semi-naturally and supporting significant biodiversity. The study of the Etruscan necropolis of Monterozzi in Italy, a UNESCO site, revealed high floristic richness and relictual vegetation features, as bioindicators of past human activities and ecological continuity, with notable biodiversity [13]. A syntaxonomic research at the Pasargadae World Heritage Site in Iran further illustrates the ecological and historical significance of vegetation communities at archaeological sites, while also signalling shifts to vegetation patterns, due to climate change, highlighting the need for adaptive conservation strategies [10].

Moreover, evidence from burial mounds confirms that passive restoration following archaeological excavations can effectively support biodiversity, landscape integrity, and the reestablishment of native plant communities, offering a valuable model for conservation planning [67]. Looking ahead, sustainable conservation demands the use of climate-resilient, low-maintenance, and locally adapted plant species. As UNESCO emphasises, building climate resilience into heritage landscapes is essential for long-term preservation [68].

The objectives of environmental sustainability are also important in light of pressures from mass tourism on archaeological sites, a critical concern in heritage management discourse. While tourism can bring economic benefits and visibility to heritage sites, unplanned or poorly managed tourism often results in physical degradation, habitat disruption, overuse of resources, and damage to Outstanding Universal Value (OUV) [69]. Integrating sustainable tourism management with landscape architecture and vegetation strategies could provide a more adaptive, resilient framework for protecting archaeological heritage under increasing visitor pressure.

The convergence of cultural and ecological approaches has given rise to the concept of the archaeological park. In recent decades, the term “archaeological park” has come to describe broader areas that unite cultural and natural heritage [70]. These parks protect heritage, support local communities, and promote sustainable tourism by integrating regional features and long-term sustainability [70,71]. Their design improves physical and intellectual access to sites [26], meeting the needs of diverse visitors [72].

In Italy, Legislative Decree 42/2004 defines cultural heritage as including both cultural and landscape assets, while Decree 18.04.2012/179 outlines three stages for establishing archaeological parks [73]. This includes a scientific study of archaeological assets and landscape features, a protection and development plan, addressing interventions, land-use, risk management, education and access, and a management plan that outlines long-term sustainability. This integrated framework emphasises heritage as part of a broader cultural and ecological system [73].

Italy has designated 18 archaeological parks, including renowned sites like Pompeii, Herculaneum, Paestum, and the Appian Way, that reflect a modern approach to heritage management. These parks integrate archaeological remains with their surrounding landscapes, balancing conservation, accessibility, and public engagement.

The Appian Way Park, Rome’s city walls, and the ArchaeoGRAB are key examples of how archaeological sites can be reimagined as active urban systems that support daily life, sustainable mobility, and biodiversity. Through an interdisciplinary design approach, these interventions highlight landscape architecture as a strategic tool for integrating cultural heritage with modern urban needs. This vision of landscaped urbanity reconnects fragmented spaces and layers historical and contemporary infrastructures into the living fabric of the city [74].

International examples further illustrate the potential of archaeological parks. In Empúries, Spain, research, educational programmes and modern technologies were integrated to enrich the site’s narrative and visitor experience [72]. In Caesarea, Israel, cultural and ecological elements combined to create an immersive visitor experience [46] (p. 355) [75].

The Rock Art Archaeological Park of Campo Lameiro in Galicia, Spain, encompasses an important collection of prehistoric petroglyphs carved into granite outcrops, depicting geometric motifs, animals and human figures. Intervention was deemed necessary because uncontrolled spontaneous vegetation—primarily shrubs and trees such as *Ulex europaeus*, *U*. *minor*, *Eucalyptus globulus*, and *Pinus pinaster*—was degrading the landscape and obscuring the petroglyphs. Guided by archaeobotanical research, invasive or inappropriate species were removed, and efforts were made to reconstruct the area’s flora between 3000 and 2000 BCE. The interventions sought to create an open, legible landscape enabling visitors to understand the engravings in relation to natural features. The park supports research, recreation and education while balancing accessibility with high conservation standards [46] (pp. 360–361).

Thoughtfully integrated vegetation transforms archaeological parks into dynamic conservation and interpretive landscapes [49,66]. Botanical planning serves both ecological and museological roles, preserving biodiversity while connecting plant life into historical identity [45]. Although individual studies have examined specific aspects of vegetation use—such as conservation performance, ecological value, or visitor comfort—there is a clear lack of publications that systematically compare different vegetation strategies in terms of their combined impact on monument preservation, ecological outcomes, and social engagement.

However, the expanded concept of archaeological parks—where vegetation plays an active narrative and protective role—remains underexplored. In Southeast Asia, archaeological sites largely lack long-term environmental management plans, and vegetation strategies are often superficial. While the World Monuments Fund’s project at Preah Khan exemplifies a thoughtful, ecologically informed approach—emphasising natural succession and selective management—most other sites neglect landscape considerations, simply remove vegetation, or adopt ornamental, urban park-like solutions. Countries like Thailand favour turf lawns, hedges, and decorative elements, and garden design may reflect foreign aesthetic traditions rather than vernacular landscapes [76].

## 5. Conclusions

The evolving role of vegetation in archaeological site conservation reflects a growing recognition of landscapes as living, dynamic components of cultural heritage. Through the lens of landscape architecture, vegetation emerges as a designed and managed medium that connects ecological processes, spatial experience, and heritage values. Rather than serving as a backdrop to the monument, vegetation becomes an integral component of the archaeological landscape’s form, function, and meaning.

Vegetation planning should enhance the monument’s visibility while harmonising with the broader environment, following principles established since the Athens Charter (1931). Plants serve as vital elements in linking monuments spatially, culturally, and visually, reinforcing the holistic character of archaeological parks. Landscape architectural approaches must protect and enhance the genius loci, maintaining the emotional and historical resonance of sites as emphasised by the Venice Charter and the Québec Declaration. Thoughtful landscape design elevates archaeological sites into multifunctional spaces for cultural, educational, ecological, and recreational engagement.

Native species and those with historical relevance must be prioritised, using archaeobotanical evidence and ancient literary sources to inform planting schemes. Spontaneous and native flora offer ecological and interpretive value, supporting biodiversity and reinforcing cultural narratives. Potential threats from vegetation must be managed through ecological evaluation, selective removal, and non-invasive methods, prioritising the monument’s physical stability. Where supported by evidence, garden reconstructions can deepen public understanding of historical landscapes, provided they remain reversible and transparent. Plants can symbolise missing architectural elements, enhancing site legibility without compromising authenticity.

Strategic vegetation planning, informed by environmental modelling, can stabilise site microclimates, protect remains, and improve visitor experience. Archaeological sites must be conceived as active nodes within living urban and ecological systems, contributing to sustainable development and community well-being. Through this lens, vegetation is not merely preserved or controlled; it is designed, interpreted, and activated as a medium of heritage expression. It bridges the past and present, nature and culture, conservation and creativity, affirming its place as a central tool in the landscape architectural treatment of archaeological heritage.

## Data Availability

No new data were created or analysed in this study. Data sharing is not applicable to this article.
